# Cytokines/Chemokines: Potential Biomarkers for Non-paraneoplastic Anti-N-Methyl-D-Aspartate Receptor Encephalitis

**DOI:** 10.3389/fneur.2020.582296

**Published:** 2020-12-21

**Authors:** Jingwen Liu, Lei Liu, Wenting Kang, Gongxin Peng, Di Yu, Qiuying Ma, Yatong Li, Yan Zhao, Lin Li, Feifei Dai, Jiawei Wang

**Affiliations:** ^1^Department of Neurology, Beijing Tongren Hospital, Capital Medical University, Beijing, China; ^2^Medical Research Center, Beijing Tongren Hospital, Capital Medical University, Beijing, China; ^3^Center for Bioinformatics, Institute of Basic Medical Sciences, Chinese Academy of Medical Sciences & School of Basic Medicine, Peking Union Medical College, Beijing, China

**Keywords:** anti-NMDAR encephalitis, cytokine, chemokine, biomarker, prognosis

## Abstract

**Objective:** Anti-N-methyl-D-aspartate receptor (NMDAR) encephalitis is the most common type of autoimmune encephalitis. This study focuses on finding new biomarkers to evaluate the clinical condition and provide new directions for treatment.

**Methods:** A total of 44 cytokines/chemokines in the cerebrospinal fluid of 10 non-paraneoplastic patients and nine controls were measured. We selected some of the cytokines/chemokines that significantly increased in patients. Six selected cytokines/chemokines, including IL-10, CXCL10, CCL22, CCL3, IL-7, TNF-α, and three previously reported (IL-2, IL-6, and IL-17A), were measured in seven other patients who provided repeat samples. We compared their levels and explored correlations with severity of disease and antibody titers.

**Results:** The levels of Th1 axis (CXCL10, TNF-α, IFN-γ, CCL3), Th2 axis (CCL1, CCL8, CCL17, CCL22), Treg axis (IL-10), Th17 axis (IL-7), and B cell axis (CXCL13) cytokines, as well as IL-12 p40 and IL-16, were significantly higher in patients compared to those in controls. The level of IL-2 was significantly decreased at the intermediate stage of treatment compared with that before treatment. The severity of disease is positively correlated with levels of CXCL10, CCL3, IL-10, CCL22, and IL-6. The level of CCL3 in the high antibody titer group was greater than that in the low antibody titer group.

**Conclusion:** The pathogenesis of anti-NMDAR encephalitis involves T cell and B cell cytokines. T cells likely assist B cells to produce antibodies. IL-2, CXCL10, CCL3, IL-10, CCL22, and IL-6 may represent new biomarkers in anti-NMDAR encephalitis. Given the lack of research on IL-10, CCL3, and CCL22 in this disease, it will be informative to explore their potential role in pathogenesis in larger studies.

## Introduction

Anti-N-methyl-D-aspartate receptor (NMDAR) encephalitis is the most common type of autoimmune encephalitis, accounting for about 80% of patients (1). Dalmau and colleagues described the clinical characteristics of anti-NMDAR encephalitis, which is caused by specific immunoglobulin G (IgG) antibodies against NMDAR on the neuron membrane (2). The antibody-mediated progressive deficiency of NMDAR and the gradual recovery of receptor function with decreased antibody titer may be the cause of aggravation and remission of the disease, respectively (3).

Clinically, antibody titer can reflect the severity of the disease, but this has some limitations. Continuous intrathecal synthesis of antibody does not necessarily indicate that encephalitis is in the active phase. In some patients, antibody titers do not change significantly when clinical symptoms are alleviated and can remain positive even after complete recovery (4, 5). Recently available indicators of neuro-inflammation are not highly sensitive, for example, MRI of the head is abnormal in only 30% of affected patients (6). Cerebrospinal fluid (CSF) markers including cytological examination, oligoclonal banding, and CSF-IgG synthesis rate also lack sensitivity and specificity.

Inflammation is associated with the development and progression of cancer, stroke, and coronary artery disease (7–9). However, little research has been done on its role in anti-NMDAR encephalitis. It has been reported that prolonged or secondary elevation of CXCL13 is associated with relapse or poor response to treatment (10). High levels of BAFF and APRIL in CSF suggest poor prognosis (11). Anti-NMDAR encephalitis is characterized by the activation of Th17-related factors IL-17 and IL-6 (12). The level of Th1-related factor CXCL10 increases in CSF in the early stage, while TNF-α and IFN-γ increase in CSF at all stages (13). In addition, tocilizumab (an IL-6 inhibitor) and low-dose IL-2 are effective in the treatment of patients with refractory autoimmune encephalitis (14, 15).

Cytokines/chemokines, as important immunoregulatory factors, play a key role in complex inflammatory reactions. Therefore, we compared the levels of T cell- and B cell-related cytokines/chemokines in the CSF of patients with anti-NMDAR encephalitis and controls and discovered cytokines/chemokines that significantly increased in the acute phase of the disease. Then, we measured some of the selected cytokines/chemokines (IL-10, CXCL10, CCL22, CCL3, IL-7, TNF-α) and IL-2, IL-6, and IL-17A in patients with anti-NMDAR encephalitis who provided CSF samples several times to find new biomarkers for clinical evaluation and to provide new directions for treatment.

## Materials and Methods

### Subjects and Samples

Firstly, 10 patients (*n* = 10) with non-paraneoplastic anti-NMDAR encephalitis were recruited to the Central Laboratory of Beijing Tongren Hospital from June 2016 to June 2018 and provided CSF. They met the following criteria: (a) diagnosis met the Graus and Dalmau criteria (16) and (b) the titer of anti-NMDAR antibody in CSF was more than 1:100–1:320. Nine patients (*n* = 9) who provided CSF were recruited to the control group. The main diagnosis of the group included cranial hypertension syndrome, intracranial venous sinus thrombosis, and pseudooptic papillary edema. Their autoimmune encephalitis antibody in CSF was negative. In addition, subjects who met the following exclusion criteria were excluded: (1) definite or suspected central nervous system infection, (2) definite or suspected neuromyelitis optica spectrum disorders or multiple sclerosis (MS), (3) definite or suspected systemic immune disease, and (4) abnormal routine CSF, biochemical, and CSF-IgG synthesis rate results. Ten samples from anti-NMDAR encephalitis patients plus nine samples from controls were collected for testing.

Subsequently, another seven patients with non-paraneoplastic anti-NMDAR encephalitis were recruited to the Central Laboratory of Beijing Tongren Hospital from February 16, 2016 to November 13, 2018, who provided CSF at different stages of the disease. Their diagnosis met the Graus and Dalmau criteria (16). Twenty-seven samples from them were collected for testing.

The studies involving human participants were reviewed and approved by the ethics committee of Beijing Tongren Hospital, Capital Medical University. The patients or their legal guardians provided their written informed consent to participate in this study. All experiments were performed in accordance with relevant guidelines and regulations.

### Methods

#### Collection and Preservation of Samples

CSF samples were collected and separated into Eppendorf tubes. The samples were frozen at −80°C and thawed no more than twice.

#### Quantitative Detection of Cytokines/Chemokines

MILLIPLEX MAP multiple biomarker detection technology was used. The HCYTOMAG-60K Human Cytokine/Chemokine Magnetic Bead Panel was used to detect 29 cytokines/chemokines including IL-10, IL-12 p40, IL-12 p70, IL-13, IL-15, IL-17A, CXCL10, CCL2, CCL7, CCL22, CCL3, CCL4, CCL11, CX3CL1, G-CSF, GM-CSF, GRO, IFN-α2, IFN-γ, IL-1β, IL-1Ra, IL-2, IL-4, IL-5, IL-6, IL-7, IL-8, CCL5, and TNF-α. HCYP2MAG-62K Human Cytokine/Chemokine Panel II was used to detect a further 15 cytokines/chemokines including CCL21, CCL27, CXCL5, CCL24, CCL26, CCL1, IL-16, IL-21, IL-23, CXCL13, CCL8, CCL13, CCL15, CXCL12, and CCL17 (Table 1).

**Table 1:** Cytokines/chemokines detected in the study.

**Magnetic bead panel**	**Th1 axis**	**Th2 axis**	**Treg axis**	**Th17 axis**	**B cell axis**	**Broad spectrum**
HCYTOMAG-60K	IFN-γ	IL-2	IL-10	IL-6		IL-12 p40
	TNF-α	IL-4		IL-7		IL-12 p70
	CXCL10	IL-13		IL-8		IL-15
	CCL3	CCL22		IL-17A		CCL2
	CCL5	CCL7		G-CSF		CCL4
		CCL11		GM-CSF		CX3CL1
						GRO
						IFN-α2
						IL-1β
						IL-1Ra
						IL-5
HCYP2MAG-62K		CCL1		IL-23	CXCL13	CCL21
		CCL8			CXCL12	CCL27
		CCL13				CXCL5
		CCL17				CCL24
						CCL26
						IL-16
						IL-21
						CCL15

*Th, helper T cell; Treg, regulatory cells*.

#### Data Analysis and Statistics

Statistical analyses were performed using SPSS 22.0 and R language. Statistical graphs were generated using GraphPad Prism 7.0. Medians and ranges were used to describe the quantitative data. Fisher's exact test was used for comparisons between frequency of categorical variables. Mann–Whitney *U* test was used for pairwise comparisons. Kruskal–Wallis test was used to compare multiple groups of samples, with Mann–Whitney *U* test for *post hoc* analysis and Bonferroni correction. Correlations between parameters were analyzed using Spearman correlation; *p* < 0.01 was considered to be significant in the comparison of samples between patients with anti-NMDAR encephalitis and controls, and *p* < 0.05 was considered to be significant in other comparisons.

## Results

### Identification of Cytokines/Chemokines That Increase in the Acute Phase of Anti-NMDAR Encephalitis

#### Clinical Data in Part One of the Study

The median age of patients with anti-NMDAR encephalitis was 27 (14–35) years, and 60% were female. The median age of controls was 38 (15–53) years, and 100% were female. There was no significant difference in age (*P* = 0.211) and gender (*P* = 0.087) between patients and controls. The detailed clinical presentations and MRI, EEG, and CSF findings are shown in Supplementary Table 1.

#### Comparison of the Levels of Cytokines/Chemokines Between Patients With Anti-NMDAR Encephalitis and Controls

**Figure 1:**
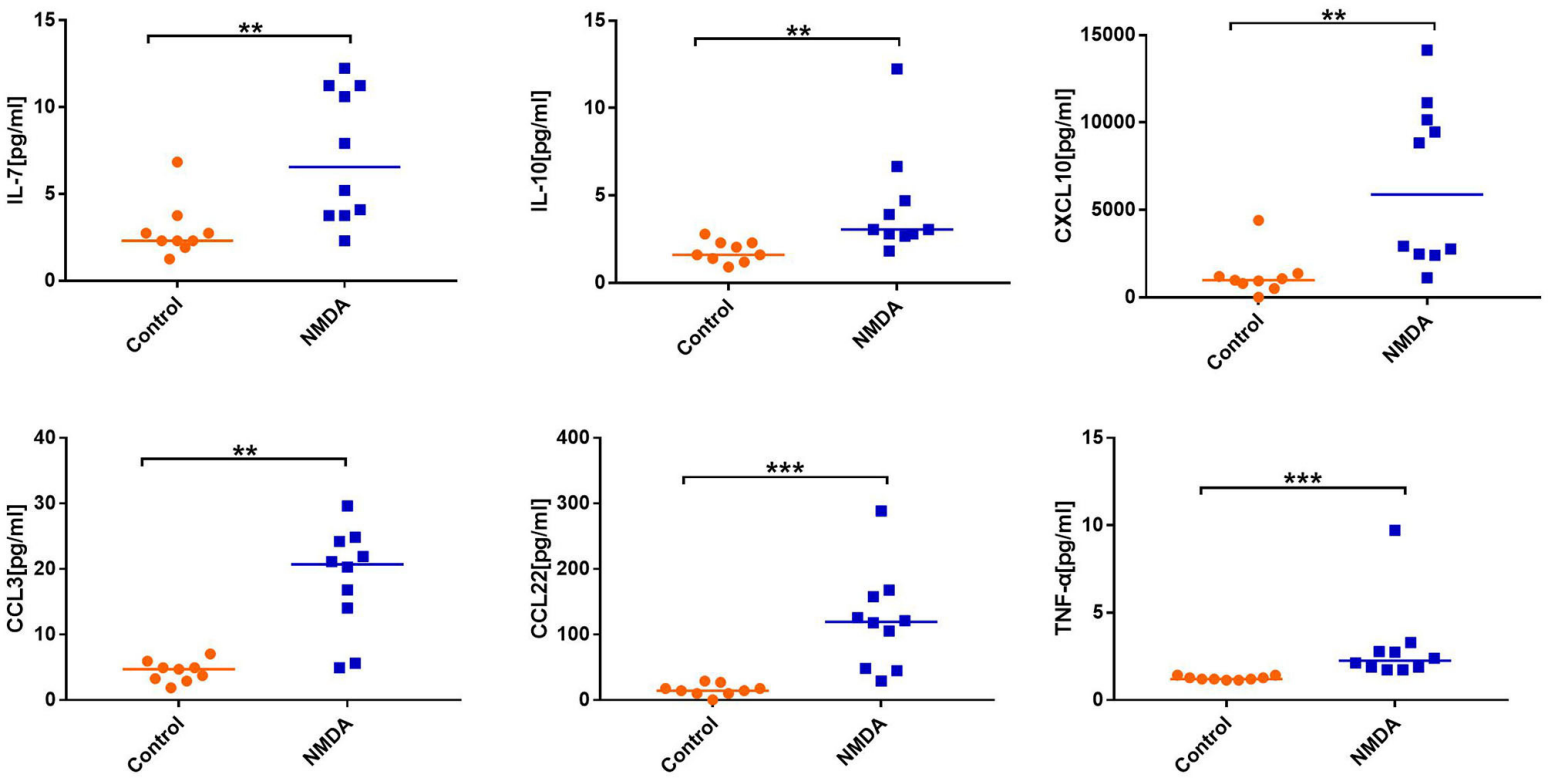
Comparison of the level of cytokines/chemokines between anti-N-methyl-D-aspartate receptor (NMDAR) encephalitis group and control group. Mann–Whitney *U* test was used. Only cytokines/chemokines that were statistically significantly different and selected for the next experiment are shown. Median cytokine/chemokine levels are indicated. **P < 0.01, ***P < 0.001. NMDA, anti-NMDAR encephalitis group; Control, control group.

Statistical analysis showed that the levels of IFN-γ (*P* = 0.001), TNF-α (*P* < 0.001), CXCL10 (*P* = 0.001), CCL3 (*P* = 0.001), CCL1 (*P* = 0.001), CCL8 (*P* = 0.007), CCL17 (*P* = 0.005), CCL22 (*P* < 0.001), IL-7 (*P* = 0.005), CXCL13 (*P* = 0.001), IL-10 (*P* = 0.002), IL-12 p40 (*P* = 0.002), and IL-16 (*P* = 0.004) in CSF were significantly increased in patients with anti-NMDAR encephalitis compared to those of controls (Figure 1).

### Longitudinal Changes in Cytokines/Chemokines in Patients With Anti-NMDAR Encephalitis

#### Clinical Data in Part Two of the Study

Four males and three females with anti-NMDAR encephalitis were recruited. Their median age was 20 (17–35) years. The median follow-up time was 15 (8–29) months. The clinical symptoms of patients at the peak of the disease included one or more of the following: abnormal behavior, seizures, speech dysfunction, involuntary movement, coma, autonomic dysfunction, hypoventilation, focal neurological lesions, and cognitive dysfunction. Four patients had changes in brain MRI and 6 in electroencephalogram. Treatment was initiated within 1.5 months from the onset of the disease in all patients and included intravenous immunoglobulin (IVIG) and/or corticosteroids. Rituximab (RTX) was used in patient 3. Patients 1, 5, 6, and 7 were treated with mycophenolate mofetil (MMF) to prevent recurrence (Table 2).

**Table 2:** Clinical data in part two of the study.

**Patient**	**Age (years)**	**Follow-up time (months)**	**Symptoms at the peak of the disease**	**MRI**	**EEG**	**Time to start treatment (days)^a^**	**Therapy^b^**
1	30–35	9	Abnormal behavior, cognitive dysfunction	Abnormal signals beside the anterior horn of the bilateral ventricles	Each lead on both sides mixed with 6–7 Hz of slow wave	9	IVIG: 2 g/kg MP: 1g/day * 3 days, then decreasing sequentially MMF: 1.5 g/day
2	20–25	29	Abnormal behavior, cognitive dysfunction, seizures, involuntary movement	Normal	A large amount of 5–7 Hz of slow waves	27	IVIG: 2 g/kg MP: 1 g/day * 3 days, then decreasing sequentially
3	16–20	8	Abnormal behavior, seizures, speech dysfunction, coma, hypoventilation, cognitive dysfunction	Abnormal signals beside the anterior horn of the bilateral ventricles	Slow wave background, reduced voltage	24	IVIG: 4 g/kg MP: 1 g/day * 3 days, then decreasing sequentially RTX: 375 mg/m^2^, once a week for 4 weeks
4	16–20	13	Seizures, involuntary movement, autonomic dysfunction, cognitive dysfunction	Multiple abnormal signals in bilateral temporal pole, insular lobe, hippocampus, and left frontal cortex	Paroxysmal, medium–high amplitude 3–7 Hz of slow wave in each lead	10	IVIG: 4 g/kg PA: 30 mg/day, then decreasing sequentially MP: 1 g/day * 3 days, then decreasing sequentially
5	16–20	15	Abnormal behavior, seizures, involuntary movement, autonomic dysfunction, hypoventilation, stiff neck, cognitive dysfunction	Normal	Moderate to severe abnormality, consistent with change in encephalitis	8	IVIG: 2 g/kg MP: 1 g/day * 3 days, then decreasing sequentially MMF: 0.5 g/day
6	26–30	23	Seizures, involuntary movement, left hand numbness	Normal	Mild abnormality	44	MP: 0.5 g/day * 3 days, then decreasing sequentially MMF: 0.25 g/day
7	16–20	20	Seizures, cognitive dysfunction	Suspected mild meningitis in both temporal regions	Normal	31	IVIG: 10 g/kg MMF: 1 g/day

a *Days of onset when anti-NMDAR encephalitis treatment was initiated*.

b *IVIG (intravenous immunoglobulin)—The dose of IVIG for a course was 2 g/kg, which was intravenously infused over 5 days; MP (methylprednisolone)—The dose of 0.5–1 g/day was continuously intravenously infused for 3 days and reduced by half every 3 days until oral administration; then, the reduction was 4 mg every 2 weeks. The total course was about 6 months; MMF (mycophenolate mofetil)—This treatment was maintained for at least 1 year. RTX (rituximab). PA (prednisone acetate)*.

**Figure 2:**
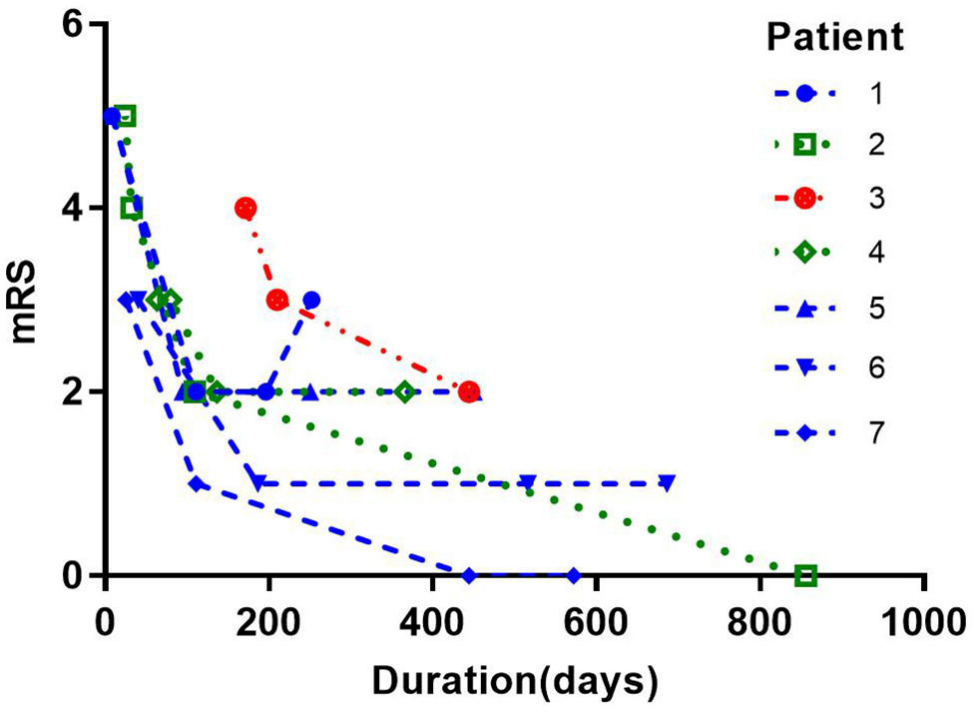
Modified Rankin score (mRS) in patients in part two of the study. Green: only first-line treatment (IVIG/corticosteroids). The median mRS was 2.5 (0–5). Blue: first-line treatment plus mycophenolate mofetil. The median mRS was 2 (0–5). Red: first-line treatment plus rituximab. The median mRS was 3 (2–4). One recurrence occurred in patient 1 during follow-up; his mRS went up after falling. The other nine patients showed a downward trend in mRS over time, and their clinical symptoms gradually improved.

Clinical condition was assessed by the modified Rankin score (mRS). The median mRS of patients with only first-line treatment (IVIG/corticosteroids), first-line treatment plus MMF, and first-line treatment plus RTX was 2.5 (0–5), 2 (0–5), and 3 (2–4), respectively. One recurrence occurred in patient 1 during follow-up; his mRS went up after falling. The other nine patients showed a downward trend in mRS over time, and their clinical symptoms gradually improved (Figure 2).

#### Change in Cytokines/Chemokines at Different Stages of Anti-NMDAR Encephalitis

Twenty-five of 27 samples were divided into three groups. Group 1 comprised patients before treatment. Group 2 comprised patients whose treatment had lasted for 2–3 months (the intermediate stage of treatment). Group 3 comprised patients whose treatment had lasted for more than 3 months (the late stage of treatment). mRS decreased gradually with treatment. The positivity rate of anti-NMDAR antibody decreased in group 2 compared to that in group 1, while it went up slightly in group 3 (Table 3).

**Table 3:** Basic data of patients with anti-N-methyl-D-aspartate receptor (NMDAR) encephalitis at different stages of the disease.

	**Samples/** **patients**	**Duration (days)^a^**	**Positivity rate of anti-NMDAR antibody**	**mRS**
Group 1	5/5	24 (8–40)	100%	5 (3–5)
Group 2	8/7	111 (63–186)	87.50%	2 (1–4)
Group 3	12/7	444 (196–856)	91.70%	2 (0–3)

a *Time from the collection of the samples to the first clinical symptoms*.

**Figure 3:**
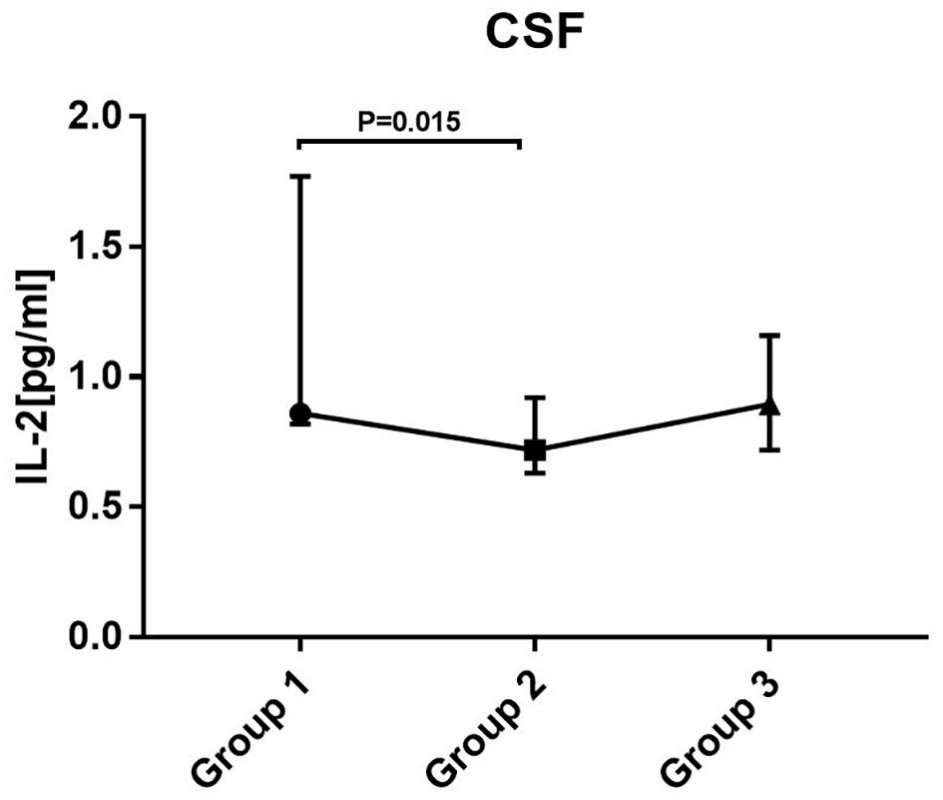
Levels of cytokines/chemokines in patients at different stages of disease. Kruskal–Wallis test with Mann–Whitney *U* test for *post hoc* analysis and Bonferroni correction were used. Only changes in IL-2 between group 1 and group 2 were statistically significant. Group 1: before treatment. Group 2: treatment had lasted for 2–3 months (the intermediate stage of treatment). Group 3: treatment had lasted for more than 3 months (the late stage of treatment).

In each group, the average was taken for patients who provided two or more samples. Statistical analysis showed that the level of IL-2 was significantly different between groups 1 and 2 (*P* = 0.015). The level of IL-2 showed a downward trend from the early to the intermediate stage of treatment and increased again at the later period (not statistically significant between groups 2 and 3). There were no significant differences in the levels of other cytokines/chemokines between groups (Figure 3).

#### Correlation Between mRS and Cytokines/Chemokines in Patients With Anti-NMDAR Encephalitis

**Figure 4:**
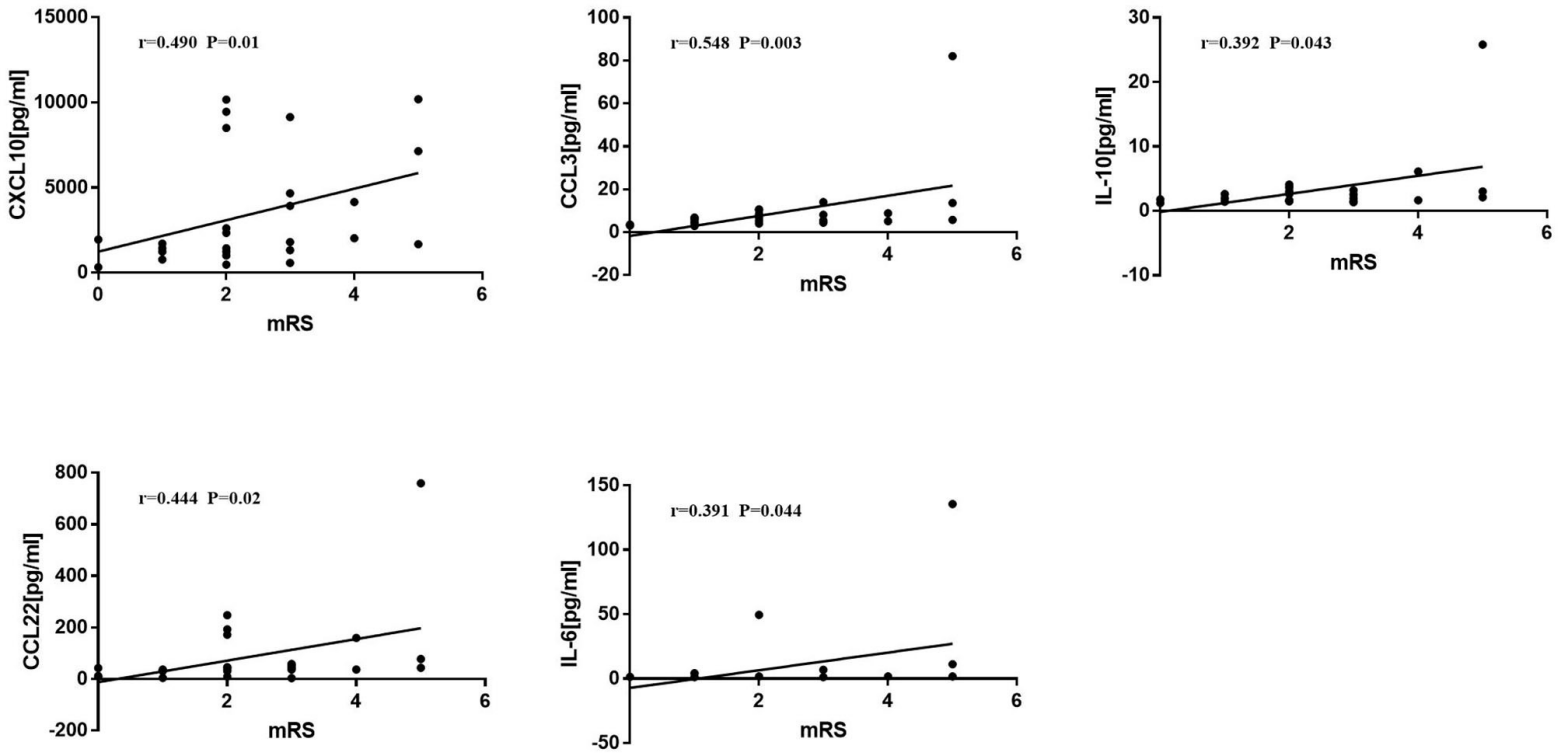
Correlations between Modified Rankin score (mRS) and cytokines/chemokines. Spearman correlation analysis was used. mRS was positively correlated with CXCL10, CCL3, IL-10, CCL22, and IL-6 (*p* < 0.05).

mRS was positively correlated with the levels of CXCL10 (*r* = 0.490, *P* = 0.01), CCL3 (*r* = 0.548, *P* = 0.003), IL-10 (*r* = 0.392, *P* = 0.043), CCL22 (*r* = 0.444, *P* = 0.02), and IL-6 (*r* = 0.391, *P* = 0.044) (Figure 4).

#### Comparison of the Level of Cytokines/Chemokines at Different Antibody Titers

**Figure 5:**
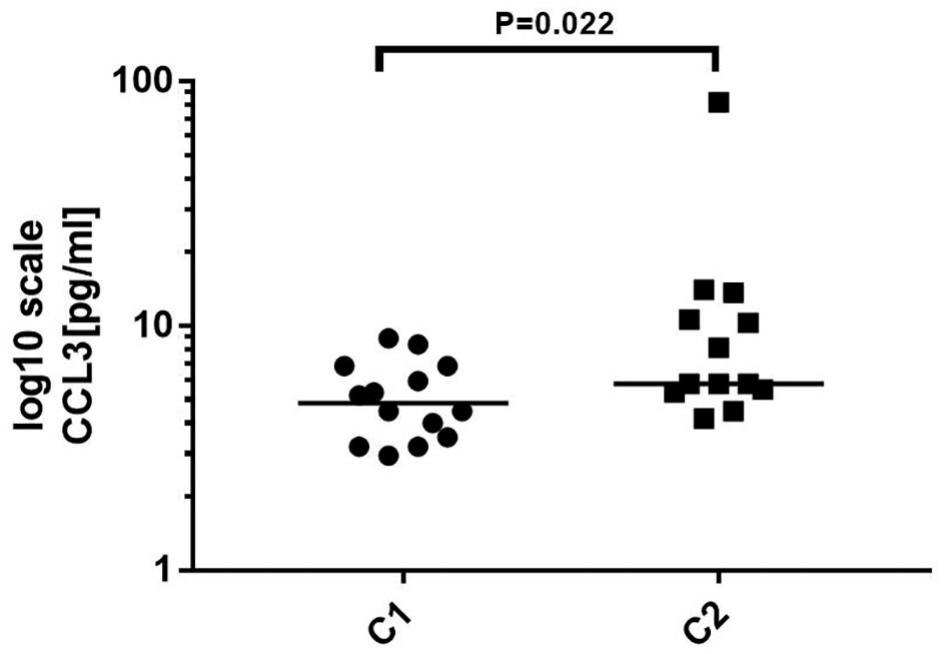
Comparison of the level of cytokines/chemokines at different antibody titers. C1: antibody titer in cerebrospinal fluid (CSF) was negative ~1:100, *n* = 14. C2: Antibody titer in CSF was more than 1:100~1:320, *n* = 13. Mann–Whitney *U* test was used. The level of CCL3 was significantly increased in C2 compared to that in C1 (*p* < 0.05).

Twenty-seven samples were divided into two groups according to antibody titer. C1 included 14 samples, and the titer ranged negative ~1:100. C2 included 13 samples, and the titer ranged more than 1:100~1:320. Statistical analysis showed that the level of CCL3 (*P* = 0.022) was significantly increased in C2 compared to that in C1. There were no significant differences in the levels of the remaining cytokines/chemokines between the two groups (Figure 5).

#### Comparison of the Level of Cytokines/Chemokines Between RTX Group and MMF Group

There were no significant differences in the levels of cytokines/chemokines between the two groups.

### Single Case Study

**Figure 6:**
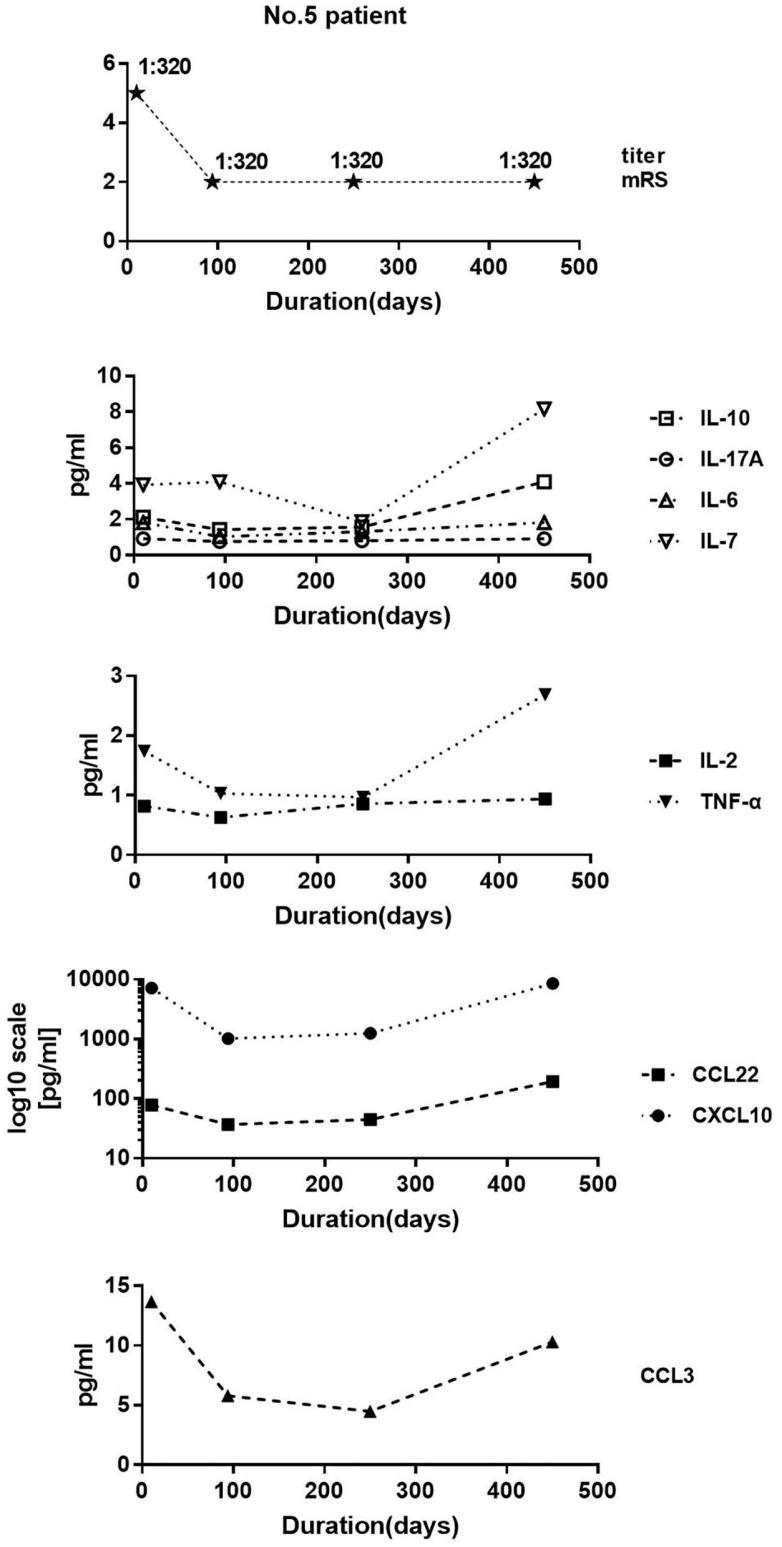
Patient 5 data. We collected four samples at 10, 94, 250, and 450 days after disease onset. Modified Rankin score at these four time points were 5, 2, 2, and 2, respectively. Antibody titer was 1:320, 1:320, 1:320, and 1:320. Clinical symptoms improved on day 94 compared with those on day 10. Despite equal antibody titers in the cerebrospinal fluid, the levels of IL-10, CCL22, IL-17A, IL-2, IL-6, CXCL10, CCL3, and TNF-α decreased.

We collected samples of patient 5 at 10, 94, 250, and 450 days after disease onset. The mRS at these four time points was 5, 2, 2, and 2 respectively. The antibody titer was 1:320, 1:320, 1:320, and 1:320. The clinical symptoms improved on day 94 compared with those in day 10. Despite equal antibody titers in the CSF, the levels of IL-10, CCL22, IL-17A, IL-2, IL-6, CXCL10, CCL3, and TNF-α decreased. Unexpectedly, all cytokine/chemokine levels in CSF on day 450 were higher than those on day 252, even if the patient's symptoms remained stable (Figure 6).

## Discussion

There has been no previous literature on anti-NMDAR encephalitis selecting cytokines/chemokines by such an extensive measurement. In the present study, the levels of cytokines associated with the Th1 axis (CXCL10, TNF-α, IFN-γ, CCL3), Th2 axis (CCL1, CCL8, CCL17, CCL22), Treg axis (IL-10), Th17 axis (IL-7), B cell axis (CXCL13), and IL-12 p40/IL-16 in anti-NMDAR encephalitis were significantly higher than in the controls. The levels of IL-6 and IL-17A, implicated in previous literature (12), did not increase significantly. It was probably that all of our patients were in the acute phase of the disease with a high antibody titer. Perhaps the balance of cytokines/chemokines makes IL-6 and IL-17A not significantly increase.

According to the introduction of immune mechanisms in Janeway's Immunobiology (17), we speculate the following immune process. IL-7 can promote the differentiation of precursor B cells and precursor T cells. Th1 and Th2 axis-related cytokines/chemokines can promote the differentiation of CD4+ T cells into Th1 and Th2. CXCL10 and CCL3 mediate the chemotaxis of Th1 cells to pathologic sites, while CCL1, CCL8, CCL17, and CCL22 mediate the chemotaxis of Th2 cells to pathologic sites and stimulate the production of associated antibodies by B cells. Th1 cells produce TNF-α to promote inflammation and IFN-γ which controls Th1 differentiation. Additionally, CXCL10 and CXCL13 can help the B cells to produce antibody. CCL17 and CCL22 can also promote the differentiation of CD4+ T cells into Treg, while Treg produces IL-10, which is able to inhibit the activation and differentiation of other immune cells, thus facilitating the recovery of patients. Thus, the pathogenesis of anti-NMDAR encephalitis involves T cells and B cells, with T cells likely to assist B cells in antibody production.

The levels of some selected cytokines/chemokines were detected in patients who provided samples several times, including molecules associated with Th1 axis (CXCL10, TNF-α, CCL3), Th2 axis (CCL22), Treg axis (IL-10), and Th17 axis (IL-7). Previous studies have shown that tocilizumab and low-dose IL-2 were effective in the treatment of refractory autoimmune encephalitis (14, 15, 18). Therefore, te levels of IL-2, IL-6, and IL-17A were also assessed.

In the present study, IL-2 decreased significantly at the intermediate stage of treatment (group 2) compared with before treatment (group 1). All the patients received first-line treatment (IVIG and/or glucocorticoids), and those in group 2 were in the first-line treatment period. IVIG and high-dose glucocorticoid pulse therapy could inhibit the production of various inflammatory cytokines/chemokines. However, their period of effectiveness was short (19), resulting in the level of IL-2 at the late stage of treatment (group 3) to increase with a reduction in glucocorticoid dosage. In addition, low-dose IL-2 can restore the balance between Treg and effector T cells in autoimmune diseases (20), which may be another reason why the level of IL-2 in group 3 went up. The results indicate that IL-2 level during treatment could be used to evaluate the therapeutic effects and the immune response status of patients. As mRS decreased gradually during the three treatment periods, the positivity rate of anti-NMDAR antibody decreased at the intermediate stage of treatment, while it went up slightly at the late stage of treatment. This phenomenon suggests that antibody titers do not necessarily reflect disease status. Therefore, we then explored the correlation between mRS and cytokines/chemokines, finding that mRS was positively correlated with CXCL10, CCL3, IL-10, CCL22, and IL-6.

CXCL10 binds to C-X-C receptor 3 (CXCR3). High levels of CXCL10 in tissue fluids are a marker of immune response, especially that related to Th1. Th1 cells recruited by CXCL10 promote the production of IFN-γ and TNF-α, which can further stimulate the secretion of CXCL10 (21) and increase the persistence of the immune response. In patients with viral encephalomyelitis, IFN-γ can induce memory B cells to express CXCR3 (22) and respond to elevated CXCL10, promoting the production of antibody in the central nervous system (CNS) (23). Previous literature has reported significantly increased CXCL10 in the active phase of rheumatoid arthritis, Sjogren's syndrome, systemic lupus erythematosus, and other autoimmune diseases (24). CCL3, which binds to C-C receptor 1 (CCR1), CCR4, and CCR5, is an activator of innate and adaptive immune responses and plays a key role in regulating lymph node homing of dendritic cells and inducing antigen-specific T cell responses. CCL3 is released in large quantities during induction of the Th1 immune response. Indeed its production is an important mechanism for maintaining cell recruitment during inflammatory responses (25). The role of CCL3 in autoimmune diseases is seldom discussed in the literature. CCL22 is the ligand of C-C cytokine receptor 4 (CCR4). Activated macrophages, associated with Th2 responses and tissue repair, can produce large amounts of CCL22 (26). Bone marrow cells can induce the production of CCL22 *via* Th2-related cytokines IL-4 and IL-13 and play a positive feedback amplification role in Th2 inflammatory reactions (27, 28). Previous literature has reported that CCL22 in the CSF of MS patients increases significantly, while natalizumab (a blocking antibody against adhesion molecule integrin α4) could reduce CCL22 (29, 30). IL-6 is essential for B cells to differentiate into plasma cells and produce antibodies (31). It can promote the production of Th2 axis cytokines IL-4 and IL-13 to enhance Th2 differentiation, while inhibiting the differentiation of Th1, and is a regulatory factor that maintains the functional balance of Th1 and Th2 (32). IL-6 promotes Th17 differentiation by activating transcription factors and can block the formation and activity of Treg, so it is also considered as a regulator, balancing Treg and Th17 (33).

Unlike the above-mentioned cytokines/chemokines, IL-10 is considered to negatively regulate the immune response process. IL-10 is an anti-inflammatory cytokine, which can restrict inflammatory and immunopathological processes in many CNS diseases. After binding with a receptor, it transmits signals which reduce the expression of cytokine/chemokine genes, restrains antigen presentation to T cells (34, 35), and enhances the expression of anti-apoptotic factors that prevent cell apoptosis. Our results indicated that the autoimmune reaction intensified and the clinical symptoms progressed with increased CXCL10, CCL3, CCL22, IL-6, and IL-10. This provides evidence supporting the effectiveness of tocilizumab (an IL-6 inhibitor) in the treatment of refractory anti-NMDAR encephalitis (14, 18). The progression of the disease stimulates the expression of IL-10 to inhibit immune inflammation and tissue damage, thus balancing various immune pathways and promoting recovery from the disease. Meanwhile, the level of these cytokines/chemokines decreased gradually with an improvement of the clinical condition, suggesting that the inflammatory immune response decreases gradually. Moreover, the level of CCL3 was significantly increased with high antibody titers, suggesting that it could relate to the synthesis of anti-NMDAR antibodies. Leypoldt et al. (10) reported that the level of CXCL13 in CSF was related to the intrathecal synthesis of anti-NMDAR antibodies.

MMF can inhibit the proliferation of T cells and B cells, while RTX targets B cells. However, there were no significant differences in the levels of cytokines/chemokines between the two treatments. B cells can contribute to MS pathogenesis by activating T cells (36). We speculate that the targeting effect of RTX on B cells also inhibits the activation of T cells in anti-NMDAR encephalitis, resulting in a decrease in T cell-related cytokines.

Patient 5 was a typical patient with no significant change in antibody titer when the clinical symptoms improved. In the early and intermediate stage of the disease, the level of most cytokines/chemokines decreased alongside the improvement of clinical symptoms, but increased unexpectedly in the later stage. The treatment of corticosteroids is usually maintained for about 6 months. We speculate that the inhibitory effect on cytokines/chemokines reduced as corticosteroids gradually withdrew, resulting in increased levels of cytokines/chemokines on day 450. This result suggests that the antibody titer in the early and the intermediate stage of the disease do not reflect the clinical situation and the therapeutic effect; cytokines/chemokines do but may not do so in the later stages of the disease.

Our study has several limitations. A smaller sample size may lead to a degree of bias. Due to the deficiency of the healthy control group in the second step, it is regrettable that the cytokine/chemokine differences between patients and healthy controls in different periods cannot be analyzed. Only some, but not all, of the selected cytokines/chemokines were measured in the second step, indicating that other potential markers might actually exist.

## Conclusion

The pathogenesis of anti-NMDAR encephalitis involves T cells and B cells. T cells are likely to assist B cells to produce antibodies. IL-2, CXCL10, CCL3, IL-10, CCL22, and IL-6 may represent new biomarkers in patients with anti-NMDAR encephalitis. Given the lack of research on IL-10, CCL3, and CCL22 in this condition, it will be important to explore their potential roles in future larger studies.

## Data Availability Statement

The raw data supporting the conclusions of this article will be made available by the authors, without undue reservation.

## Ethics Statement

The studies involving human participants were reviewed and approved by the ethics committee of Beijing Tongren Hospital, Capital Medical University. Written informed consent to participate in this study was provided by the participants' legal guardian/next of kin.

## Author Contributions

The experiment design was performed by LLiu and JW. Data analysis was performed by JL, LLiu, and GP. The first draft of the manuscript was written by JL. Antibodies testing was performed by LLiu and WK. The cytokine/chemokine measurement was performed by WK. Manuscript revision and direct patient care were performed by all the authors.

## Conflict of Interest

The authors declare that the research was conducted in the absence of any commercial or financial relationships that could be construed as a potential conflict of interest.
