# Supplementary material for: Cytokines/Chemokines: Potential Biomarkers for Non-paraneoplastic Anti-N-Methyl-D-Aspartate Receptor Encephalitis
Source: Front Neurol. 2020 Dec 21;11:582296. doi: 10.3389/fneur.2020.582296 (PMC7779630; doi:10.3389/fneur.2020.582296)
Supplement: Supplementary file 1 [file Table_1.pdf]

Supplementary Material

1 Supplementary Table

Supplementary Table S1 Clinical features of subjects in part one of the study

Patient	Age (years)	Sex	symptoms	Infection	CSF				MRI	EEG
					Leukocyte (*10^6/L)	Chloride (mmol/L)	Glucose (mmol/L)	Protein (mg/dl)		
1	18	female	Abnormal behavior, seizures, involuntary movement, left hand numbness	None	2	121.2	3.46	35.4	Normal	A few 4-7Hz slow waves in each lead on both sides
2	29	female	Abnormal behavior, seizures, speech dysfunction, ataxia	None	20	128.2	3.26	47.6	Abnormal signals in left frontal lobe and bilateral brain stem	Each lead on both sides mixed with 6-7Hz slow wave
3	31	male	Abnormal behavior, cognitive dysfunction, involuntary movement, hypoventilation, autonomic dysfunction, coma	Pneumonia, urinary tract infection	36	120.3	2.98	49.8	Abnormal signals in bilateral temporal lobe and hippocampus	A large amount of 4-6Hz slow waves in each lead on both sides, reduced voltage
4	27	female	Abnormal behavior, cognitive dysfunction	None	6	129.8	4.48	23.2	Abnormal signals beside bilateral anterior horn of ventricles	A large amount of 3-6Hz slow waves in each lead on both sides
5	27	male	Seizures, involuntary movement	None	14	138	3.32	12.1	Normal	A few slow waves in bilateral frontal leads

6	30	male	Abnormal behavior, cognitive dysfunction, seizures, involuntary movement, amaurosis	None	10	120.4	3.28	19.8	Normal	Paroxysmal, medium-high amplitude 4-7Hz slow wave in each lead.
7	35	male	Abnormal behavior, speech dysfunction, autonomic dysfunction	None	32	124.8	2.92	27.5	Abnormal signals in left frontal lobe, brain stem and bilateral peripheral ventricles	Moderate abnormality
8	23	female	Abnormal behavior, seizures, cognitive dysfunction	None	8	122.5	3.19	17.2	Normal	A few slow waves in each lead on both sides
9	19	female	Abnormal behavior, seizures	Pneumonia	26	117.2	3.62	48.6	Abnormal enhancement in bilateral temporal meninges	Mild abnormality
10	14	female	Seizures, autonomic dysfunction	None	2	130.5	2.87	47.2	Normal	A few slow waves in each lead on both sides
